# Transition From Sublexical to Lexico-Semantic Stimulus Processing

**DOI:** 10.3389/fnsys.2020.522384

**Published:** 2020-10-30

**Authors:** Frederick Benjamin Junker, Lara Schlaffke, Christian Bellebaum, Marta Ghio, Stefanie Brühl, Nikolai Axmacher, Tobias Schmidt-Wilcke

**Affiliations:** ^1^Department of Neuropsychology, Ruhr-University Bochum, Bochum, Germany; ^2^Department of Clinical Neuroscience and Medical Psychology, Heinrich Heine University, Düsseldorf, Germany; ^3^Department for Neurology, Professional Association Berufsgenossenschaft-University Hospital Bergmannsheil, Bochum, Germany; ^4^Institute of Experimental Psychology, Heinrich Heine University, Düsseldorf, Germany; ^5^St. Mauritius Therapy Clinic, Meerbusch, Germany; ^6^Department of Neurology, Rheinisch-Westfälische Technische Hochschule (RWTH) Aachen University, Aachen, Germany; ^7^Division of Neuroscience and Experimental Psychology, University of Manchester, Manchester, United Kingdom

**Keywords:** sublexical processing, lexico-semantic processing, phonological lexicon, semantic system, lexicality, default mode network

## Abstract

Resembling letter-by-letter translation, Morse code can be used to investigate various linguistic components by slowing down the cognitive process of language decoding. Using fMRI and Morse code, we investigated patterns of brain activation associated with decoding three-letter words or non-words and making a lexical decision. Our data suggest that early sublexical processing is associated with activation in brain regions that are involved in sound-patterns to phoneme conversion (inferior parietal lobule), phonological output buffer (inferior frontal cortex: pars opercularis) as well as phonological and semantic top-down predictions (inferior frontal cortex: pars triangularis). In addition, later lexico-semantic processing of meaningful stimuli is associated with activation of the phonological lexicon (angular gyrus) and the semantic system (default mode network). Overall, our data indicate that sublexical and lexico-semantic analyses comprise two cognitive processes that rely on neighboring networks in the left frontal cortex and parietal lobule.

## Introduction

Understanding language is one of the most demanding human abilities, involving different processing stages, ranging from primary perceptual analysis to semantic and syntactic integration (Friederici, 2002). Investigating these stages with appropriate experiments remains challenging. Using Morse code (MC), we recently established a reading-type learning paradigm that allowed us to investigate certain stages of language processing within a highly controlled learning environment (Schmidt-Wilcke et al., 2010; Schlaffke et al., 2015). Within this framework, we could show that lower perceptual and higher lexico-semantic processing of MC relies on a common network consisting of the left premotor cortex, the supplementary motor area (SMA) and the inferior parietal lobule (IPL). While perceptual processing engaged a stronger activation in the superior temporal gyrus and SMA, lexico-semantic processing showed higher activation in the left inferior frontal cortex (IFC) and middle temporal gyrus (MTG), regions that are critically involved in language processing (Friederici, 2011). In the current project, we used MC to get further insights into lexico-semantic processing and to study a specific type of access to the lexicon, and probably to the semantic system, by non-lexical processing.

**FIGURE 1:**
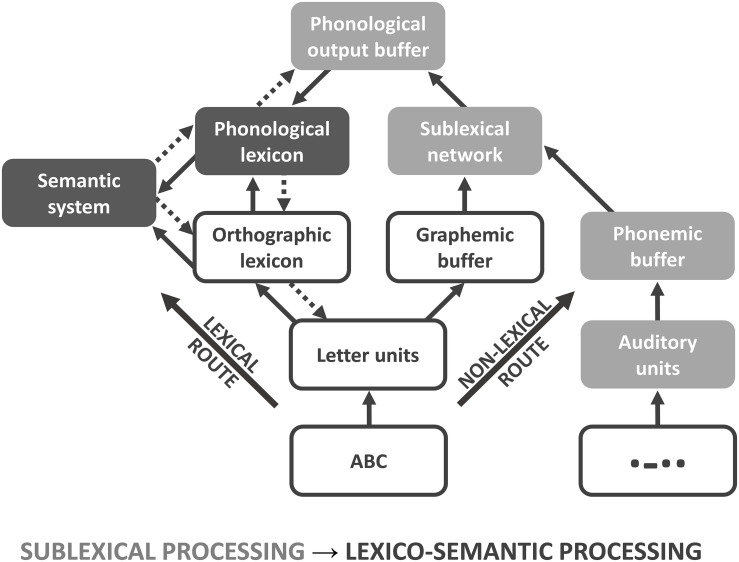
Connectionist dual process (CDP+) model. Connectionist dual process (CDP+) model for processing written language, adapted for Morse code (MC). Because of the serial presentation and learning procedure, MC words and non-words can only be processed via the non-lexical route. For analysis, the model is subdivided into sublexical (light gray) and lexico-semantic (dark gray) processing phases.

Various neurocognitive models have been established describing the processing of written (encoded) language at different processing stages, e.g., the triangle model (Seidenberg and McClelland, 1989), the dual route cascade (DRC) model (Coltheart et al., 2001), and the connectionist dual process (CDP+) model (Perry et al., 2007). All models provide two routes, both of which can be used for articulation and understanding of single words in healthy subjects (Levy et al., 2009) as well as in patients with alexia (Rapcsak et al., 2007) and dyslexia (Bergmann and Wimmer, 2008; Ziegler et al., 2008). In the triangle model, orthography (encoded language) is either directly mapped to phonology (spoken language), or indirectly mapped via semantic representations (Harm and Seidenberg, 2004). A separation between sub- and whole-word mappings is not implemented in the triangle model. In contrast, the DRC model provides information about sublexical (sub-word) and lexical (whole-word) processing stages. The lexical route maps the orthography (whole-word) of known words (orthographic lexicon) either directly to their corresponding phonology (phonological lexicon) or indirectly via the semantic system (Coltheart et al., 2001). In contrast, the grapheme-phoneme system of the non-lexical route contains rules for converting single graphemes (sub-words) into phonemes. In the CDP+ model (Figure 1), the lexical route is identical to the DRC model (Perry et al., 2007). In contrast to the DRC model, the grapheme-phoneme conversions within the sublexical system are not restricted to single grapheme-phoneme mappings. Instead, it requires cipher knowledge, i.e., statistical relationships to map graphemes on phonemes, which are transiently stored and assembled within the phonological output buffer (Zoril, 2010). Both, the orthographic (target of the lexical route) and the phonological lexicon (target of the non-lexical route) interact with the semantic system, which is critical for attributing a concept to a previously identified word. While the lexical route is essential for decoding irregular words, the non-lexical route is required for decoding pseudowords (Taylor et al., 2013). Both routes enable the correct pronunciation and understanding of encoded language like written symbols; however, the lexical route is considered to be faster, especially in skilled readers.

Various paradigms have been used to probe these two routes separately, in order to unravel the underlying functional neuroanatomy and, furthermore, to identify their interactions with the lexicon and the semantic system. So far, functional brain imaging studies have suggested explicit roles for specific brain regions (for a review, see Jobard et al., 2003; Taylor et al., 2013). While the left IPL/SPL is particularly involved in grapheme-phoneme conversions (sub-word processing; e.g., Mayer et al., 2016), the left occipitotemporal cortex and fusiform gyrus can be related to orthographic processing (whole-word processing; e.g., Glezer et al., 2009) and the orthographic lexicon (e.g., Kronbichler et al., 2007). For the phonological output buffer and for mapping orthography to semantics, a participation of the IFC is likely (e.g., Friederici, 2002; Liakakis et al., 2011). The activation of semantic representations by processing words can be related to activation in the precuneus/posterior cingulate cortex and the angular gyrus (Wirth et al., 2011; Taylor et al., 2013), especially for words with high degrees of semantic information (Graves et al., 2016).

Although functional magnetic resonance imaging (fMRI) and positron emission tomography (PET) have greatly advanced our understanding of the functional neuroanatomy underlying language (in particular word) comprehension, more work needs to be done to further disentangle the neural correlates of these different linguistic components in terms of a dynamic process. The processing speed of single words (approximately 300–500 ms) and the low temporal resolution of functional neuroimaging methods, including fMRI, pose an unsolved problem in this respect. Studies using high temporal resolution methods, such as magnetoencephalography (MEG) and electroencephalography (EEG), have suggested specific temporal order effects (Vartiainen et al., 2009); e.g., for written language, early potentials reflect orthographic (200 ms) and phonological (250 ms) processing (Holcomb and Grainger, 2007), while semantic processing takes place around 400 ms (Kutas and Hillyard, 1980). This temporal signature of speech processing is likely to be related to the spread of information from primary sensory cortices to higher association cortices. Beside these stimulus-driven bottom-up processes (Figure 1: straight arrows), there are also strong arguments for an additional top-down modulation during different processing stages (Figure 1: doted arrows; for review, see Carreiras et al., 2014). Most work has been done with respect to orthographic processing within the left occipitotemporal cortex and fusiform gyrus. Here, a modulation through phonological (Perea et al., 2016) and semantic properties (Whaley et al., 2016; Evans et al., 2017) was found that facilitates performance and neuronal responsiveness (Strijkers et al., 2015). In addition, intrinsic connection between sublexical (sub-word processing within SPL) and orthographic representation (whole-word processing within fusiform gyrus) were found to be modulated by task-intention (Deng et al., 2012), that is not reflected in current models of language decoding (like CDP+; see Figure 1). However, more work needs to be done to spatially map this spread of information onto specific brain regions.

MC has several advantages, specifically in the way letters are learned (i.e., to decode only single MC letters) and presented (i.e., in a strictly consecutive way that requires an initial sound pattern-to-phoneme conversion (sublexical processing) and a transient information storage within the phonological output buffer, without an orthographic lexicon). First, the translation of MC resembles symbol-by-symbol decoding; i.e., the auditory input is processed via the non-lexical route for both words and non-words, with the primary auditory cortices serving as cortical input channels. Secondly, the sublexical analysis is relatively slow, such that it becomes possible to disentangle the neural correlates of different processing stages via fMRI.

Using fMRI and MC (specifically words and non-words consisting of three letters), we sought to unravel the spread of information from the primary sensory cortices to higher association cortices, which reflect different stages of processing. We hypothesized that no differences between words and non-words would be found at early stages, while the letters were still being presented and translated. Common activation of the two conditions (words and non-words) could be attributed to early processing stages such as sound-pattern to phoneme conversion and phonological assembly (operationalized as sublexical processing). We then aimed to detect differences in brain activation elicited by words as compared to non-words, due to differences in their lexical and possibly semantic work up. Differences in brain activation at later stages of the processing stream, shortly after the presentation of the third letter (i.e., during lexical decision making and button press) could then be attributed to the identification of a correlate in the phonological lexicon and possibly to further semantic processing (lexico-semantic processing).

## Materials and Methods

MC is a method of encoding text information as a series of on-off tones, clicks, or lights. The International MC encodes the basic Latin alphabet, some extra Latin letters, the Arabic numerals, and a small set of punctuation and procedural signals as standardized sequences of short and long signals, which are pictured as dots (•) and dashes (—). The duration of a dash is three times the duration of a dot.

### Subjects

Thirty-four healthy, right-handed subjects (mean age 24 years, standard deviation = 2.9, 15 females) participated in the study. The data of 17 subjects were taken from the study previously published by Schlaffke et al. (2015), in which the participants learned to decipher MC letters while passively listening. Another 17 subjects had participated in a follow up study (Schlaffke et al., 2017) in which exactly the same study design was used (see below). However, participants of this second cohort in addition learned to apply MC actively during their training (5 min per session). While the analysis of these previous investigations were focused on changes in functional (Schlaffke et al., 2015) and anatomical (Schlaffke et al., 2017) connectivity involved in MC learning, the current analysis examined the neural basis of language (MC) decoding. All subjects demonstrated normal or corrected-to-normal vision. Furthermore, all participants were tested for a normal hearing frequency range of 20–20,000 Hz and none of the participants exhibited hearing impairment. The study was conducted in accordance with the Declaration of Helsinki and was approved by the Ethics Committee of the Faculty of Psychology at Ruhr-University Bochum, Germany. To be enrolled in the study, participants provided written informed consent.

### Training Procedure

The training procedure as well as the experimental task (see next section) have been described in detail elsewhere (Schlaffke et al., 2015), and here we report only the details relevant for the current analysis. All participants were naive to MC prior to the learning intervention. Using an audiobook, a subset of 12 MC letters were learned in six training sessions on 6 separate days (day 1: E, S, N, and O; day 2: T and R; day 3: U and D; day 4: A and I; day 5: M and G; day 6: repetition of all letters) in a standardized procedure. The audio book was played on stereo headphones by Philips (40 mm speaker, 20–20,000 Hz frequency range, 98 dB sensitivity, 32 Ohm impedance, 500 mV maximum power input). Study participants learned 12 MC letters in a specific order, with one learning session lasting approximately 30 min. From the second day of training, a repetition of the previously learned MC letters was performed, followed by the practice of two new letters and the decoding of three-letter MC-trains. The participants of the second cohort additionally practiced the transmittance of 30 MC letters (single letters) instead of decoding 30 acoustically presented letters, as the subjects of the first cohort did. Both groups thus followed a very similar learning protocol and, importantly, spent the same amount of time on training. The training was completed within 10 days, with an adjournment of one weekend.

### Task

All participants underwent two fMRI sessions, one before the first training session and one after the last training session. MC was presented acoustically in trains of three letters making up a word (mean stimulus length: 3.57 s), a non-word (mean stimulus duration: 3.56 s), or the SOS signal (mean stimulus duration: 2.28 s). In addition to single-letter graphemes, words and non-words included multi-letter graphemes (e.g., “ei”- [aĬ]; 11 multi-letter graphemes out of 79 different bigrams) that were not trained explicitly. Overall, 40 words and 40 non-words were presented, along with 25 SOS signals and 25 control tones (beep tone, duration: 3 s). All stimuli were produced at 786 Hz to be clearly distinguishable from scanner noise. Auditory stimulation was delivered through mri-compatible headphones at a sound level that was clearly hearable for each individual subject. Stimulus order was randomized by using the stimulus delivery and experiment control software Presentation^®^ (Neurobehavioral Systems, Albany, CA, United States). Participants were required to perform a lexical decision task after the presentation of MC stimuli. More specifically, study participants had to decode the stimuli, and then decide whether the three letters comprised a word or a non-word. Subjects responded via button press with the left hand (button 1: word; button 2: non-word). Responses with two other buttons (3 and 4) were required when the SOS signal or the control tone were presented. The time window between stimulus offset and button press is used as reaction time and represents the time-window for later lexico-semantic analysis. In addition to the lexical decision task, a perceptual task was also performed using the same stimulus material (not subject to the current analyses).

### Behavior

Since MC is used only rarely as a model for language decoding, behavioral parameters were analyzed. First, recognition performances and reaction times (RT) between words and non-words were compared using *t*-tests. Second, the influence of various stimulus properties on performance and RT were investigated using multiple linear regressions. For words, the effect of stimulus duration and frequency, based on a German word corpus including literature from 2000 to 2010, is tested. For non-words, stimulus duration and Levenshtein-distance, defined as the number of changes required to transform a non-word into a word (Yarkoni et al., 2008), were used as independent variables.

### fMRI Sequences

Magnetic Resonance Imaging was performed on a 3.0 Tesla scanner (Philips Achieva 3.2, Best, Netherlands), using a 32-channel head coil. First, high-resolution T1-weighted data sets (TR 8.3 ms, TE 3.8 ms, field of view 256 × 256, yielding 220 transversal slices with a voxel size of 1.00 × 1.00 × 1.00 mm^3^ and reconstructed to 0.94 × 0.94 × 1.00 mm^3^) were acquired from all subjects. During the actual task, T2^∗^-weighted echo planar imaging (single shot EPI with a 90° flip angle, TR 2400 ms, TE 35 ms, FOV 224 × 224 mm^2^ with a voxel size of 2 × 2 × 3 mm^3^ yielding 36 slices in an ascending scan order without gaps) produced 246 dynamic scans per run. Each MRI session (pre, post) consisted of two runs per task (lexical decision task/perceptual task—not discussed here) and required approximately 40 min (Task × Run × scans × TR).

### fMRI Preprocessing and First Level Analyses

Functional images were converted from DICOM to NIfTI (HDR-IMG pairs) format using MRIconvert 2.0 (Lewis Center for Neuroimaging, University of Oregon, United States). Pre-processing of functional images was performed using the SPM12 (Statistical Parametric Mapping) software (Welcome Department of Cognitive Neurology, University College London, London, United Kingdom) running under Matlab R2017a. The preprocessing steps included slice-time correction, unwarping, realignment for movement correction, co-registration to the structural T1-image, spatial normalization to the same stereotactic space (using the SPM EPI-template), and spatial smoothing (full width at half maximum: 6 mm).

**FIGURE 2:**
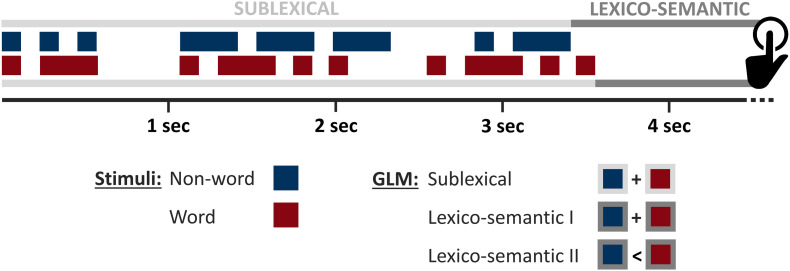
Experimental design. Time domains involved in sublexical (light gray) and lexico-semantic (dark gray) processing of specific words (red; here: “all”) and non-words (blue; here: “soa”), presented as Morse code (MC). Squares representing short stimuli (dots), rectangles long stimuli (dashes). Letters are separated by large white spaces. The feedback of the subjects was performed with the left hand (black).

First level analyses were performed in the statistical framework of the general linear model, implemented in SPM 12. To examine sublexical processing (SL), where MC letters were analyzed, decoded, and assembled, the time point of the initial stimulus presentation was determined for each stimulus (i.e., stimulus onset + duration/Figure 2: light gray). The lexico-semantic analysis (LS) was initiated after the presentation of the last letter. During this period, the previously identified and assembled phonemes were used to perform the lexical decision. Accordingly, the lexico-semantic phase was defined as the time between stimulus offset and button press (Figure 2: dark gray). For both periods, one regressor (each with the respective onset times) was created for words, non-words, SOS signals and control tones, separately for correct and incorrect identified stimuli. These, together with information about the durations of the time windows of interest (see above), were convolved with the hemodynamic response function. Importantly, the latter analysis was restricted to those stimuli that were correctly classified. During the first-level analysis, all relevant conditions (described above) were tested against the same implicit baseline. However, since these conditions were then contrasted in the second level analyses, the implicit baseline does not have an effect on the later results. Furthermore, the six movement parameters (three rotation parameters and three translation parameters) were added as covariates of no interest. Overall, first level analyses yielded six contrast images for correctly identified stimuli (i.e., SL_words, SL_nonwords, SL_control, LS_words, LS_nonwords, LS_control). Of note, brain activity related to SOS decoding was not subject to this analysis.

### fMRI Second Level Analyses

Second level analyses were performed for correctly identified stimuli (words, non-words, control tone) within a flexible factorial design (stimulus type and processing phase as factors with age as a nuisance variable). While a first evaluation of the behavioral data revealed an effect of sex upon reaction-times for non-words (*r* = −0.374; *p* = 0.029), sex was included as additional nuisance variable. Voxel-wise whole brain analyses were performed with an initial significance level of *p* < 0.001 (voxel level); the resulting clusters were deemed significant after correction for multiple comparisons (family wise error correction: *p* < 0.05 at the cluster level). Labeling was performed using the SPM12 extension Automated Anatomical Labeling^1^; the visualization was performed using the SPM12 extension bspmview^2^. The following analyses were performed:

**Analysis 1a**—**Sublexical processing I**

To identify the brain regions involved in sublexical (SL) processing of words and non-words, the following conjunction analysis was performed: (SL_words > SL_control) ^ (SL_nonwords > SL_control).

**Analysis 1b**—**Sublexical processing II**

To identify the brain regions more involved in decoding and recognizing words than non-words (and vice versa) in the sublexical phase, the following two independent analysis were performed: SL_words > SL_nonwords (1), and SL_words < SL_nonwords (2).

**Analysis 2a**—**Lexico-semantic processing I**

To identify the brain regions involved in lexico-semantic (LS) processing of words and non-words, the following conjunction analysis was performed: (LS_words > LS_control) ^ (LS_nonwords > LS_control).

**Analysis 2b**—**Lexico-semantic processing II**

To identify the brain regions more involved in decoding and recognizing words than non-words (and vice versa) in the lexico-semantic phase, the following two independent analysis was performed: LS_words > LS_nonwords (1), and LS_words < LS_nonwords (2). Furthermore, an access to their semantic meaning is possible for words only.

**Analysis 3a**—**Sublexical vs. Lexico-semantic processing I**

To identify the brain regions more involved in sublexical than lexico-semantic processing of words and non-words (and vice versa), the following two independent analysis were performed: (SL_allwords > SL_control) > (LS_allwords > LS_control) (1), and (SL_allwords > SL_control) < (LS_allwords > LS _control) (2).

**Analysis 3b**—**Sublexical vs. Lexico-semantic processing II**

To identify the brain regions more involved in decoding and recognizing words than non-words in the sublexical phase as compared to lexico-semantic phase (and vice versa), the following two independent analysis were performed: (SL_words > SL_nonwords) > (LS_words > LS_nonwords) (1), and (SL_words > SL_nonwords) < (LS_words > LS_nonwords) (2).

## Results

### Behavioral Data

After training, the lexical decision task was performed with an average accuracy of 73.5% across words and non-words [standard error of mean (SEM): 2.2%]. Non-words were correctly identified (accuracy 87.9%, SEM 2.3%) more frequently [*p* < 0.001, *F*_(1, 33)_ = 0.287] than words (accuracy 59%, SEM 2.7%). Additionally, there was a significant difference [*p* < 0.001, *F*_(__1, 33)_ = 0.79] in RT between recognized words (2073 ms, SEM 62.2 ms) and non-words (2441 ms, SEM 65.1 ms). Recognition performances were significantly correlated with RT for words (*p* < 0.001, *r* = −0.544), but not for non-words (*p* = 0.805, *r* = −0.04). Multiple linear regression analysis (see Table 1) revealed an effect of non-word duration (*p*_var_ = 0.048, *r*_var_ = −0.293) and Levenshtein-distance (*p*_var_ = 0.011, *r*_var_ = −0.381) on RT (*p*_model_ = 0.002), but not on performance (*p*_model_ = 0.265). A similar effect of words duration and frequency was not found, neither for performance (*p*_model_ = 0.685) nor RT (*p*_model_ = 0.616). Systematic errors, such as wrong translation of specific letters in all stimuli, were not observed in any subject.

**TABLE 1:** Behavior.

		Performance			Reaction time		
		p_model_	p_var_	r_var_	p_model_	p_var_	r_var_
Words	Duration	0.685	0.977	–0.005	0.616	0.328	–0.161
	Frequency		0.388	–0.142		0.946	–0.011
Non-words	Duration	0.265	0.114	–0.263	0.002	0.048	–0.293
	Levenshtein-distance		0.479	0.116		0.011	–0.381

*Multiple linear regressions for testing the effect of word duration and frequency, as well as non-word duration and Levenshtein distance on behavioral performance and reaction time. The p-value per model (p_model_), as well as the p-values (p_var_) and standardized correlation coefficient (r_var_) for each independent variable is given.*

### fMRI Data

**Analysis 1a**—**Sublexical processing I**

**FIGURE 3:**
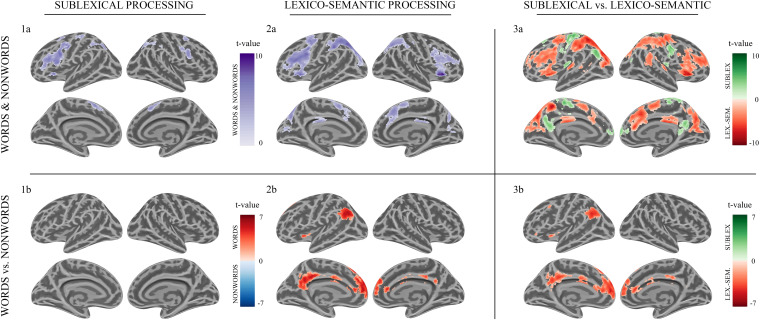
Morse code processing. Statistical parametric maps of cortical brain activation for sublexical (1a, 1b) and lexico-semantic (2a, 2b) processing of Morse code (MC) after learning. Voxel significance at *p* < 0.001, corrected for multiple comparisons at the cluster level.

Sublexical processing of words and non-words elicited brain activity in the superior and inferior frontal cortex (including pars triangularis and opercularis), the sensory-motor cortex (supplementary, precentral, paracentral), the inferior parietal lobule (IPL) as well as in the insular cortex, putamen, and caudate nucleus (Figure 3: 1a). For further information regarding peak coordinates, cluster extensions, and area activation (see Table 2).

**TABLE 2:** Sublexical processing (Analysis 1a).

	Sublexcial processing					
	Words and non-words > Control					
	Refers to analysis 1a					
	L/R	x	y	z	*z*-value	Voxel #
Precentral cortex	L	–50	–4	47	8.33	1,614
Precentral cortex	L	–44	8	29	8.25	
Inferior frontal cortex (Triangularis)	L	–44	26	23	7.28	
Supplementary motor area	L	–6	12	56	7.74	602
Supplementary motor area	L	–2	2	68	7.36	
Supplementary motor area	R	8	14	50	6.47	
Caudate nucleus	L	–10	12	–1	7.55	390
Insula cortex	L	–28	24	2	7.38	
Putamen	L	–18	8	–1	6.72	
Precentral cortex	R	42	4	29	6.07	377
Precentral cortex	R	54	–2	41	4.88	
Inferior parietal lobule	L	–28	–66	41	5.22	294
Inferior parietal lobule	L	–40	–46	47	4.35	
Inferior parietal lobule	L	–34	–48	41	4.31	
Inferior parietal lobule	R	36	–46	41	4.71	263
Angular gyrus	R	28	–64	44	4.67	
Angular gyrus	R	30	–58	38	4.14	
Caudate nucleus	R	12	12	2	6.66	261
Thalamus	R	2	–6	5	3.79	
Caudate nucleus	R	20	24	–1	3.60	
Precentral cortex	L	–34	–28	62	5.92	195
Postcentral cortex	L	–28	–24	50	4.16	
Superior frontal cortex	L	–24	0	53	5.55	149
Superior frontal cortex	R	26	0	47	5.32	103

*MNI-coordinates (x, y, z) of peak voxels and corresponding z-values of significant clusters (p < 0.001; FWE-corrected on cluster-level) for early sublexical processing of words and non-words. Additionally, cluster size (in number of voxels) and hemisphere (L = left; R = right) are shown.*

**Analysis 1b**—**Sublexical processing II**

During sublexical processing, activation patterns elicited by words and non-words did not differ significantly (Figure 3: 1b).

**Analysis 2a**—**Lexico-semantic processing I**

Lexico-semantic processing of words and non-words elicited brain activity in the inferior frontal cortex (IFC), the left precentral gyrus, the left superior parietal lobule (SPL) and IPL, the insular cortex, as well as in the middle cingulate cortex and caudate nucleus (Figure 3: 2a). For further information regarding peak coordinates, cluster extensions and area activation (see Table 3).

**TABLE 3:** Lexico-semantic processing (Analysis 2a).

	Lexico-semantic processing					
	Words and non-words > Control					
	Refers to analysis 2a					
	L/R	x	y	z	*z*-value	Voxel #
Insula cortex	L	– 36	20	– 4	11.60	4,607
Precentral cortex	L	– 42	4	29	8.69	
Supplementary motor area	L	0	10	53	8.37	
Superior parietal lobule	L	– 24	– 72	47	8.75	2,858
Inferior parietal lobule	L	– 30	– 58	47	8.35	
Inferior parietal lobule	L	– 40	– 40	44	8.13	
Insula cortex	R	32	26	– 1	10.37	1,994
Inferior frontal cortex (Opercularis)	R	56	16	35	6.77	
Precentral cortex	R	46	8	29	6.49	
Angular gyrus	R	32	– 60	47	6.55	1,463
Inferior parietal lobule	R	32	– 48	41	5.88	
Inferior parietal lobule	R	46	– 36	47	5.77	
Middle cingulate cortex	L	– 4	– 22	29	4.88	135
Middle cingulate cortex	R	4	– 18	26	4.75	
Middle cingulate cortex	R	6	– 28	26	4.54	
Caudate nucleus	L	– 16	– 2	14	5.73	110
Superior frontal cortex	R	32	– 4	56	4.02	89
Middle frontal cortex	L	38	0	62	3.62	
Calcarine sulcus	L	– 4	– 78	11	4.19	81
Calcarine sulcus	L	– 16	– 76	11	3.70	

*MNI-coordinates (x, y, z) of peak voxels and corresponding z-values of significant clusters (p < 0.001; FWE-corrected on cluster-level) for later lexico-semantic processing of words and non-words. Additionally, cluster size (in number of voxels) and hemisphere (L = left; R = right) are shown.*

**Analysis 2b**—**Lexico-semantic processing II**

**FIGURE 4:**
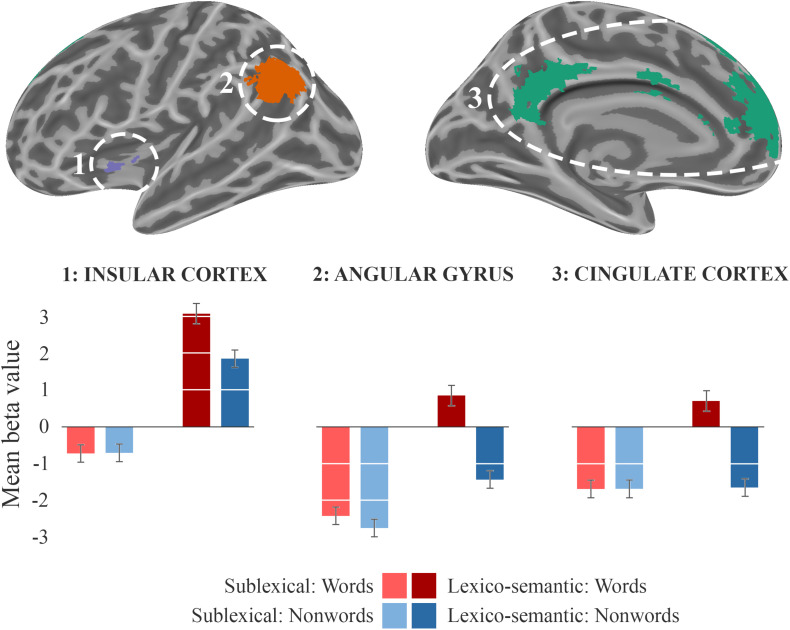
Beta-values. Beta-value analysis of clusters from analysis 2b (LS_words > LS_nonwords) for all four conditions. The corresponding mean beta values across each cluster of the sublexical processing are shown in bright colors, the lexico-semantic processing in dark colors, words in red, and non-words in blue. The standard error of the mean is displayed as error-bar.

During the lexico-semantic phase, words elicited significantly more activation than non-words in the left superior and inferior frontal cortex, the left angular gyrus and middle temporal cortex, the left insular cortex, as well as in the cingulate (anterior, middle) cortex and precuneus (Figure 3: 2b). Importantly, when looking at the mean beta-values, these regions displayed an overall deactivation during early sublexical processing for words and non-words, while only word processing leads to an activation above baseline during later lexico-semantic processing (Figure 4). No brain regions demonstrated more activation for non-words than words (Analysis 4b). For further information regarding peak coordinates, cluster extensions and area activation (see Table 4).

**TABLE 4:** Lexico-semantic processing (Analysis 2b).

	Lexico-semantic processing					
	Words > Non-words					
	Refers to analysis 2b					
	L/R	x	y	z	*z*-value	Voxel #
Anterior cingulate cortex	L	– 4	48	14	7.28	3,680
Superior frontal cortex	L	– 2	48	23	6.36	
Anterior cingulate cortex	L	– 4	40	26	6.10	
Angular gyrus	L	– 40	– 64	35	5.78	778
Middle temporal cortex	L	– 52	– 66	20	3.90	
Insular cortex	L	– 38	12	– 7	4.77	164
Insular cortex	L	– 40	4	– 4	4.05	
Inferior frontal cortex (Orbitalis)	L	– 48	26	– 7	3.75	

*MNI-coordinates (x, y, z) of peak voxel and corresponding z-values of significant clusters (p < 0.001; FWE-corrected on cluster-level) for later lexico-semantic processing of words, relative to non-words. Additionally, cluster size (in number of voxels) and hemisphere (L = left; R = right) are shown.*

**Analysis 3a**—**Sublexical vs. Lexico-semantic processing I**

Compared to lexico-semantic processing, sublexical processing of words and non-words elicited stronger brain activity in the left frontal (superior and middle) and insular cortex, the sensory-motor cortex (precentral, paracentral, postcentral), the left angular gyrus, the precuneus and cingulate cortex, as well as in the left putamen, caudate nucleus and hippocampus (Figure 3: 3a). In contrast, lexico-semantic processing of words and non-words elicited stronger brain activity in the frontal (superior, inferior) and parietal cortex (superior, inferior), the superior temporal and sensory-motor cortex (supplementary, precentral, paracentral), the right insular cortex, the cingulate cortex (anterior, middle) and precuneus, as well as in the left putamen and caudate nucleus. For further information regarding peak coordinates, cluster extensions and area activation (see Tables 5, 6).

**TABLE 5:** Sublexical vs. lexico-semantic processing (Analysis 3a).

	Sublexical > Lexico-semantic processing					
	Words and non-words > Control					
	Refers to analysis 3a					
	L/R	x	y	z	*z*-value	Voxel #
Paracentral cortex	L	– 2	– 32	50	6.35	1,053
Precentral cortex	L	– 34	– 28	59	6.17	
Postcentral cortex	L	– 26	– 26	47	6.03	
Anterior cingulate cortex	R	24	36	8	6.66	693
Superior frontal cortex	L	– 2	68	11	6.13	
Superior frontal cortex	L	– 14	66	11	5.39	
Rolandic operculum	R	40	– 18	26	6.24	542
Precentral cortex	R	44	– 12	38	6.16	
Insula cortex	R	26	– 26	26	5.50	
Thalamus	R	12	– 32	8	5.09	358
Precuneus	R	8	– 54	17	5.03	
Precuneus	L	– 6	– 52	14	4.83	
Angular gyrus	L	– 42	– 72	41	6.57	242
Angular gyrus	L	– 50	– 62	44	3.62	
Paracentral cortex	R	6	– 30	74	5.19	242
Precuneus	R	6	– 48	71	4.70	
Precentral cortex	R	14	– 32	65	4.57	
Putamen	L	– 20	18	– 1	6.11	235
Putamen	L	– 24	12	– 7	5.38	
Caudate nucleus	L	– 16	26	5	6.70	
Hippocampus	L	– 28	– 40	14	5.09	182
Cingulate cortex	L	– 16	– 46	20	4.65	
Calcarine sulcus	L	– 30	– 58	14	4.33	
Middle frontal cortex	L	– 26	20	47	4.08	91
Superior frontal cortex	L	– 20	20	56	3.71	
Middle frontal cortex	L	– 24	28	53	3.54	
Insula cortex	L	– 38	– 22	23	4.06	76
Insula cortex	L	– 38	– 16	14	3.77	

*MNI-coordinates (x, y, z) of peak voxels and corresponding z-values of significant clusters (p < 0.001; FWE-corrected on cluster-level) for early sublexical processing of words and non-words relative to later lexico-semantic processing of words and non-words. Additionally, cluster size (in number of voxels) and hemisphere (L = left; R = right) are shown.*

**TABLE 6:** Lexico-semantic vs. sublexical processing (Analysis 3a).

	Sublexical < Lexico-semantic processing					
	Words and non-words > Control					
	Refers to analysis 3a					
	L/R	x	y	z	*z*-value	Voxel #
Middle occipital cortex	L	– 28	– 74	23	8.58	4,460
Precuneus	L	– 8	– 50	50	8.06	
Superior parietal lobule	L	– 30	– 52	59	7.84	
Insula cortex	L	– 38	20	– 7	11.77	2,719
Precentral cortex	L	– 36	– 2	50	6.33	
Superior frontal cortex	L	– 28	– 4	68	6.12	
Insula cortex	R	34	26	– 4	8.24	1,957
Inferior frontal cortex (Opercularis)	R	46	18	– 1	7.63	
Insula cortex	R	30	20	8	7.55	
Precentral cortex	R	42	– 18	56	6.01	1,618
Postcentral cortex	R	46	– 24	50	5.26	
Angular gyrus	R	32	– 60	47	4.86	
Middle cingulate cortex	R	10	20	38	7.00	1,306
Anterior cingulate cortex	R	6	38	17	6.66	
Supplementary motor area	L	0	10	53	6.13	
Middle cingulate cortex	R	4	– 18	26	6.12	188
Middle cingulate cortex	R	4	– 28	29	5.82	
Middle cingulate cortex	L	– 4	– 22	29	5.38	
Caudate nucleus	L	– 16	– 2	14	6.05	155
Putamen	L	– 24	4	11	4.38	
Superior temporal cortex	L	– 42	– 32	8	5.31	145
Supramarginal gyrus	R	56	– 42	23	4.64	127
Henschl gyrus	R	48	– 20	5	5.59	98
Henschl gyrus	R	42	– 26	11	4.27	

*MNI-coordinates (x, y, z) of peak voxels and corresponding z-values of significant clusters (p < 0.001; FWE-corrected on cluster-level) for later lexico-semantic processing of words and non-words relative to early sublexical processing of words and non-words. Additionally, cluster size (in number of voxels) and hemisphere (L = left; R = right) are shown.*

**Analysis 3b**—**Sublexical vs. Lexico-semantic processing II**

Compared to sublexical processing, words elicited significantly more activation than non-words during lexico-semantic processing in the left superior frontal cortex, the cingulate cortex (anterior, middle, posterior) and the left angular gyrus (Figure 3: 3b). For further information regarding peak coordinates, cluster extensions and area activation (see Table 7).

**TABLE 7:** Lexico-semantic vs. sublexical processing (Analysis 3b).

	Sublexical < Lexico-semantic processing					
	Words > Non-words					
	Refers to analysis 3b					
	L/R	x	y	z	*z*-value	Voxel #
Anterior cingulate cortex	L	– 2	48	11	6.51	1,762
Superior frontal cortex	L	– 4	64	11	5.58	
Anterior cingulate cortex	L	– 2	38	26	5.20	
Angular gyrus	L	– 50	– 60	32	4.72	366
Angular gyrus	L	– 40	– 62	35	4.45	
Middle cingulate cortex	L	– 4	– 36	35	4.75	342
Posterior cingulate cortex	L	– 8	– 50	29	4.15	
Vermis	L	– 2	– 54	5	3.91	
Superior frontal cortex	L	– 6	44	50	4.89	223
Superior frontal cortex	L	– 10	34	50	4.81	
Superior frontal cortex	L	– 14	30	56	4.09	
Middle cingulate cortex	L	– 2	4	32	3.97	98
Anterior cingulate cortex	R	4	10	23	3.64	
Middle cingulate cortex	R	0	– 6	29	3.50	

*MNI-coordinates (x, y, z) of peak voxels and corresponding z-values of significant clusters (p < 0.001; FWE-corrected on cluster-level) for later lexico-semantic processing of words relative to non-words, compared to early sublexical processing of words relative to non-words. Additionally, cluster size (in number of voxels) and hemisphere (L = left; R = right) are shown.*

## Discussion

Using MC as a model to probe the non-lexical route and to slow down the cognitive process of word recognition, we sought to disentangle the neural correlates of two specific linguistic components (for further details, see limitations), i.e., sound-pattern to phoneme conversion and phonological assembly (sublexical analysis), as well as the neural correlates of lexical decision making (lexico-semantic analysis).

At the behavioral level, a negative correlation between non-words RT and Levenshtein-distance fit well with the effect of lexicality commonly observed (Yap et al., 2015). An effect of Levenshtein-distance on behavior stating that lexical processing performance depends on memory, where stimuli that are more distinct can be recognized faster than stimuli with higher similarities to other memory entries. Differences in recognition performance between words and non-words can be explained probabilistically. In combinations of three letters, the MC letters used here allow to build up to 89 different German words and 1,639 non-words. On average, this leads to an 18-fold greater chance for a translation error to transform MC sequence into a non-word than into a word. This leads to a bias in recognition performance between non-words and words that is typically not found when using written language.

The imaging data during early sublexical processing, where single MC letters were analyzed, decoded, and assembled, revealed brain activation in the IPL, the SMA and premotor cortex, as well as in the left IFC (i.e., Broca’s area) and insular cortex (Figure 3: 1A). In the later lexico-semantic phase, where subjects still decode the last MC letter of words and non-words, followed by phonological assembly and lexical decision, additional brain activity was observed in the frontal cortex bilaterally as well as in the parietal cortex, more pronounced on the left side (Figure 3: 2a).

The SMA and left premotor cortex have frequently been reported to exhibit activation during word reading and writing. It is conceivable that brain activation reflects concomitant sensorimotor representations of letters (Premotor: Flowers et al., 2004; SMA: Vinci-Booher and James, 2016), possibly because of the close link between reading and writing during learning (Longcamp et al., 2003) that is also existing in our learning procedure. In the current study, an activation of the left premotor cortex and SMA during both processing phases (sublexical and lexico-semantic) might indicates, that the processing of individual MC letters in unexperienced decoders does not rely on a direct MC-to-phoneme conversion (further discussed below). Instead, MC letters could be translated into graphemes first, before a phoneme association is carried out, involving representations of written letters. Still, MC decoding is highly similar to reading. Furthermore, brain activation can also be related to speech articulation at the level of letters and words (Premotor: Ghosh et al., 2008; SMA: Alario et al., 2006), as well as speech motor control during language processing (SMA: Hertrich et al., 2016). Interestingly, deaf readers have been reported to show more activation in the left premotor cortex when performing a phonological decision task, in comparison with readers with normal hearing (Emmorey et al., 2013), whereas no differences in brain activation were present when both groups were performing a semantic decision task. Increased activation during the phonological decision task was interpreted to reflect greater reliance on articulatory phonological codes. In addition to letter representation, SMA and prefrontal activation indicate that individual letters might be spoken out silently during decoding.

In addition to the left-lateralized frontal activation, that is specific to language processing, the bilateral extension during later lexico-semantic processing of words and non-words might be related to an overall more demanding processing phase (Yang et al., 2009). During this phase, the previously decoded MC letters still have to be kept in mind, assembled and compared to the phonological lexicon for final decision-making, leading to a more sophisticated processing phase.

Strong activations were also observed in the left IPL, reaching into the superior parietal lobule. The left parietal lobe is considered to serve as a major language area, critically involved in various aspects of both perception and generation of encoded language (Brownsett and Wise, 2010; Conant et al., 2014), such as grapheme-phoneme conversion and phoneme generation (Bitan et al., 2007; Du et al., 2014). With respect to the CDP+ model, which served as a conceptual framework to the current study, the sublexical system (grapheme-phoneme conversion), implemented within the non-lexical route, is essential to read either regular words (when read letter-by-letter) or pseudowords; however, the brain regions hosting these computations have not yet been fully elucidated (Levy et al., 2009). Protopapas et al. provided evidence for the involvement of the left IPL in grapheme-phoneme conversion (Protopapas et al., 2016). This is consistent with other studies suggesting a role for the left parietal lobule in the non-lexical route and grapheme-phoneme conversion. By analyzing 35 studies related to reading, Cattinelli et al. (2013) identified two separate clusters in the left IPL dependent on the stimulus material. While words elicited stronger activation in the angular gyrus (see below), reading pseudoword engaged an anterior cluster in the IPL, indicating a stronger reliance on grapheme-to-phoneme conversions within the sublexical network. Likewise, the left IPL is more engaged when subjects perform a writing-to-dictation task on pseudowords compared with words (DeMarco et al., 2017). Interestingly, in developmental dyslexia, impaired phonological processing is also associated with hypoactivity of the left IPL (Paz-Alonso et al., 2018). Importantly, we observed a remarkable spread of brain activation within the IPL and SPL toward the medial parietal cortex and the parietal operculum. While the early IPL/SPL activation reflects sound pattern to phoneme conversion, which is also necessary during later lexico-semantic processing of words and non-words and has been described above, the current study does not allow an further interpretation of the activation spread within the parietal lobule. Further studies, involving methods with high temporal resolution might be necessary for an adequate interpretation.

Other important regions that were activated during sublexical and lexico-semantic processing of words and non-words were the left IFC (pars opercularis and pars triangularis), including Broca’s area. Performing a lexical decision requires a comparison with phonologically (or orthographically) similar words within the mental lexicon. Furthermore, words elicit associations based on previously acquired knowledge of the world (i.e., an interaction with the semantic system). These findings are consistent with other brain imaging studies that have investigated the lexicality effect (Lin et al., 2016). The left IFC has been implicated in a number of tasks related to language comprehension, such as phonological output buffer (Cattinelli et al., 2013; Taylor et al., 2013), semantic as well as syntactic processing (Friederici, 2002; Makuuchi et al., 2009; Wirth et al., 2011). In a meta-analysis involving 485 neuroimaging studies, Liakakis and colleques were able to divide the IFG into functional sub-units. While the pars opercularis can be related to working memory and the phonological output buffer (Liakakis et al., 2011), the pars triangularis is involved in phonological and semantic processing during word recognition (Heim et al., 2009), where dorsal and ventral portions being related to phonological and semantic processing, respectively (Vigneau et al., 2006). In the current study, the left IFC being involved in sublexical as well as lexico-semantic processing steps possibly plays an important role in phonological assembly of converted phonemes within the phonological output buffer (pars opercularis). Additionally, an involvement of the pars triangularis already during early sublexical processing of words and non-words might indicate a modulation from phonological as well as semantic components by top-down predictions. Future studies using methods with higher temporal resolutions, such as EEG and MEG, are needed in order to attribute top-down processes in a more fine-grained manner. Connectivity analyses will also help to create neurocognitive models that describe the direction of information flow and help to distinguish bottom-up from top-down processes more precisely (Levy et al., 2009; Xu et al., 2015), which both play an important role in speech perception (Carreiras et al., 2014) and are likely to dynamically change depending on the individual’s level of expertise.

Comparing the activity elicited by words against non-words during lexico-semantic processing, significantly more activation was observed in the ventral part of the left IFC/insular cortex, the medial prefrontal cortex (anterior cingulate cortex/superior frontal cortex), the precuneus (referred to as the retrosplenial area for the remainder of this text) and the left angular gyrus (Figure 3: A4). Importantly, these differences in brain activations were observed in the lexico-semantic phase, but not in the sublexical phase (Figure 4), indicating a relevance of these regions in phonological retrieval (phonological lexicon) and linkage to semantics that is possible for words only. Nevertheless, an involvement of phonological and semantic components during early sublexical processing is still feasible for words and non-words via top-down predictions, as concluded for the IFG (pars triangularis).

Activation of the angular gyrus during reading is frequently observed in children, while skilled adult readers are missing this activation (Seghier, 2013). In contrast to less experienced readers, whose word comprehension relies primarily on non-lexical processing involving the phonological lexicon (Figure 1: non-lexical route), skilled readers are able to directly map orthography on semantics without involvement of the phonological lexicon (Figure 1: lexical route). However, one might argue that activation of the left angular gyrus can also be related to semantic processing (van Ettinger-Veenstra et al., 2016). By analyzing 36 studies related to reading, Taylor et al. (2013) concluded, that the left angular gyrus is involved in storage and retrieval of phonological and semantic information, while a further dissociation of lexical and semantic processing is not possible because of their co-occurrence. Similar effects were found for the left IFG (pars triangularis; see above), that is strongly connected to the angular gyrus by the superior longitudinal fasciculus III (Frey et al., 2008). While a distinction of phonological and semantic representations within the angular gyrus might be possible based on anatomical, functional and connectivity properties (for review, see Seghier, 2013), the current study design is not suited to make these rather fine-grained functional assignments. At the current stage, we suggest that the activation seen in the left angular gyrus possibly reflects an interaction between the phonological lexicon and the semantic system during language decoding of meaningful stimuli.

Semantic processing refers to the cognitive act of accessing stored conceptual knowledge and underlies our comprehension of word meanings (Binder et al., 2009). At a neural level, it has not yet been fully elucidated where and how externally presented stimuli interact with the semantic system. The network enabling the storage and retrieval of semantic information has been the focus of psychological and neuroscientific research for many years (Daselaar et al., 2009; Tomasello et al., 2017; Xu et al., 2017). Performing a meta-analysis to locate the semantic system, Binder et al. (2009) identified a left-lateralized network comprising seven regions, which could be further subdivided into three categories: the posterior heteromodal/multimodal association cortex, the heteromodal prefrontal cortex, and the medial limbic regions (Binder et al., 2009). With respect to the retrosplenial area, previous authors have emphasized the strong and reciprocal connections that it exhibits with the medial temporal lobe (Kobayashi and Amaral, 2007; Buckner et al., 2008), making it a candidate region to interact with the hippocampus during episodic memory formation (Daselaar et al., 2009). As such, the retrosplenial area might serve as an interface between semantic retrieval and episodic encoding, thus computing perceptual, semantic, and affective representations during an episode, while the hippocampus binds these cortical events into a unique event configuration (Buckner et al., 2008; Binder et al., 2009).

From a resting state perspective, the medial prefrontal cortex, the retrosplenial area, and the IPL, comprising the angular gyrus, are part of the default mode network (DMN; Buckner et al., 2008), which has been linked to a number of higher cognitive processes, such as semantic and autobiographical memory, prospection, and theory of mind reasoning. By examining the involvement of episodic and semantic memory, Kim (2016) demonstrated that, in the context of episodic memory, the DMN contributes more to recollection/familiarity effects than to old/new effects, and that for semantic memory it contributes more to word/pseudoword effects than to semantic/phonological effects. Overall episodic and semantic retrieval processes involving strong memory signals were shown to recruit overlapping DMN regions (Spreng and Grady, 2010; Kim, 2016). As such, the DMN supports common aspects involved in simulating internalized experiences. Importantly, apart from its role in the processing of lower level semantic units (i.e., words), the DMN seems to be critically involved in the analysis of sentences, as well as narratives (Dehghani et al., 2017). As outlined above, the DMN, also often referred to as the task-negative network, appears to be active when individuals are engaged in stimulus-independent thought. Vice versa, the DMN gets deactivated while processing external stimuli, as observed during early sublexical processing of words and non-words as well as later lexico-semantic processing of non-words. It has been proposed that this network represents an “internal world,” comprising symbolically transformed experiences (Andrews-Hanna, 2012). Metaphorically speaking, one might suggest that a word that is part of the mental lexicon serves as a door into the semantic system, which is then able to link this specific word with a variety of associations that reflect past experiences and/or accumulated world knowledge. We propose that the left insular cortex, since it was activated during sublexical and lexico-semantic processing of words and non-words; and showed a lexicality effect, serves as a functional hub by coordinating higher-order cognitive aspects during language decoding. While this functional heterogeneity is also described in the literature (e.g., Oh et al., 2014; Uddin et al., 2017), we postulate that the insular cortex might allow the switch between the task-positive (1a, 2a) and task-negative network (2b: DMN) during decoding of meaningful stimuli. One of the main challenges in future studies will be to describe this interaction on a functional level more accurately.

## Limitations

Some limitations of this study need to be mentioned. We were not able to fully control, whether study participants used exclusively the non-lexical route of the CDP+ model. One might argue, that all MC stimuli were translated mentally into graphemes first, before they were then analyzed lexically. Indeed, it might be more plausible, that the subjects performed an MC-grapheme conversion first, before converting the translated grapheme into their corresponding phoneme. This additional processing step would not just reduce the memory load within the phonemic buffer because of the simpler and more familiar structure of a single letter (as compared to sequences of short and long tones). It would also be necessary for those associations involving multi-letter phonemes (e.g., “ei”- [aĬ]), that were not trained during the learning procedure (single MC letter conversions). Therefore, a processing via the lexical route is theoretically possible. As previous studies could show (e.g., Price and Edwards, 2012), the serial presentation of individual visual letters of a word leads to a much slower processing speed (single letter reading: ∼60 words/min; whole word reading: ∼200 words/min). This indicates that the lexical route, going along with a significant increase in processing speed, is less involved in processing serially presented stimuli. In addition, highly experienced MC decoders can show an involvement of the orthographic lexicon during processing, as reflected by activation of the left fusiform gyrus/occipitotemporal cortex (Maier et al., 2004), which was not found for unexperienced decoders in the current study. Although different interpretations were possible (e.g., Perrone-Bertolotti et al., 2014), we believe that the lexical route is still negligible for serial MC processing in unexperienced decoders.

The lexicality effect and its neural correlates are known to be influenced by a number of variables, such as RT (Yarkoni et al., 2009), word frequency, and neighborhood density. These variables and their effects on neural activity require further analysis in future studies. Because of the characteristics of our learning paradigm (12 letters in 6 days) and the anticipated limitations of the phonological output buffer in predicting difficulties with words consisting of ≥3 letters, our stimulus material is currently confined to 89 possible words and 1,639 possible non-words (letter combinations); notably, this will pose challenges to the analyses of these additional aspects.

We use the rather broad term lexico-semantic analysis. Lexical decision making refers to the ability to identify a word in the mental lexicon, a task which can be performed without associating any further conceptual knowledge with the stimulus (semantic analysis). It is highly likely that such semantic processes take place when subjects identify a word, and semantic processing might even precede (to some degree) lexical decisions. However, our experiment in its current version does not allow to accurately assess the degree of semantic processing. Against this background, an additional important extension will be to not only investigate the lexicality effect, but also to implement specific semantic judgements (e.g., concrete vs. abstract and living vs. non-living).

## Conclusion

By using Morse code, we were able to disentangle different phases of language decoding in a way that the processing stages, which follow one after the other, could be studied despite the low temporal resolution of the fMRI. Our data suggest that the sublexical and lexico-semantic analyses are two distinct processes that rely on neighboring networks in the frontal cortex and parietal lobule. Early sublexical and later lexico-semantic processing is associated with activation in brain regions that are implicated in the sound-pattern to phoneme conversion (IPL), involving sensorimotor letter representations (SMA, premotor cortex), followed by phonological assembly within the phonological output buffer (IFC: Pars opercularis). Furthermore, top-down modulation by phonological and semantic predictions (IFC: Pars triangularis) are likely to occur already at early processing stages during language decoding. Later lexico-semantic processing of meaningful stimuli is additionally associated with activation in brain regions that are implicated in the phonological lexicon (angular gyrus), as well as in the activation of the semantic system (DMN). More specifically, whereas encoded language (graphemes, MC or any other code) is initially an external stimulus that undergoes analysis and processing within the task-positive network, the resulting word then gains access to the task-negative network (DMN), possibly by an involvement of the left insula cortex. In this way, semantics could be an interlink between these networks.

## Data Availability Statement

The datasets generated for this study are available on request to the corresponding author.

## Ethics Statement

The studies involving human participants were reviewed and approved by the Ethics Committee Ruhr University Bochum. The patients/participants provided their written informed consent to participate in this study.

## Author Contributions

FJ was involved in performing the analyses and drafted the manuscript. LS was involved in the setup of the paradigm and data collection. CB was involved in the interpretation of the imaging data and manuscript writing. MG was involved in the interpretation of the imaging data and proofreading. SB was involved in the analyses and interpretations of the linguistic data and performed proofreading. NA was involved in the development of the study design and the setup of the paradigm. TS-W developed the study concept, was involved in the imaging analyses, manuscript writing, and proofreading. All authors contributed to the article and approved the submitted version.

## Conflict of Interest

The authors declare that the research was conducted in the absence of any commercial or financial relationships that could be construed as a potential conflict of interest.

## Footnotes

1 http://www.gin.cnrs.fr/en/tools/aal-aal3/

2 http://www.bobspunt.com/bspmview/

## Abbreviations

CDP+ model: Connectionist Dual Process model
DMN: Default Mode Network
DRC model: Dual Route Cascade model
EEG: Electroencephalography
fMRI: functional Magnetic Resonance Imaging
FWE: Family wise Error
IFC: Inferior Frontal Cortex
IPL: Inferior Parietal Lobule
LS: Lexico-semantic
MC: Morse code
MEG: Magnetoencephalography
MNI: Montreal Neurological Institute
MTG: Middle Temporal Gyrus
MVPA: Multi-voxel pattern analysis
RT: reaction time
SEM: Standard error of mean
SPL: Superior Parietal Lobule
SL: Sublexical
SPM: Statistical Parametric Mapping
SMA: Supplementary Motor Area.
